# Effect of age on upper limb, neck, and trunk kinematics during activities of daily living

**DOI:** 10.1016/j.heliyon.2023.e20535

**Published:** 2023-10-02

**Authors:** Jun Nakatake, Hideki Arakawa, Maeda Shogo, Koji Totoribe, Etsuo Chosa

**Affiliations:** a Rehabilitation Unit, University of Miyazaki Hospital, Kiyotake-cho Kihara 5200, Miyazaki, 889-1692, Japan; b Department of Orthopaedic Surgery, Faculty of Medicine, University of Miyazaki, Kiyotake-cho Kihara 5200, Miyazaki, 889-1692, Japan; c Department of Rehabilitation, Miyazaki City Tano Hospital, Tano-cho Minamibaru 1-6-2, Miyazaki, 889-1704, Japan

**Keywords:** Biomechanics phenomena, Upper body, Self-care, Ergonomics, Motion analysis

## Abstract

Motion analysis during activities of daily living has been conducted in numerous studies. However, information is lacking regarding age-related differences that affect clinical assessment and treatment goals. This study aimed to examine the effect of age on kinematics during activities of daily living. Three-dimensional motions of the shoulder, elbow, neck, and trunk of 12 younger adults (age, 29.8 ± 5.4 years; 7 men and 5 women) and 10 older adults (age, 69.5 ± 4.9 years; 6 men and 4 women) were measured during the acts of reaching for a table, bringing a glass to the mouth for drinking, wiping the buttocks, tying shoelaces, washing hair, washing the axilla, reaching for a high shelf, and reaching for the floor. The ranges of motion and sequential joint angles were compared between age groups by using discrete analysis and statistical parametric mapping, respectively. The ranges of motion of all joint angles in older and younger adults were comparable in the drinking, washing hair, washing the axilla, and reaching for the floor tasks. Statistical parametric mapping indicated that older adults had significantly poorer neck extension than did younger adults during the drinking (67–92% cycle time) and tying shoelaces (64–95% cycle time) tasks. Kinematics were mostly maintained in healthy older adults during activities of daily living. However, reduced motions were confirmed later during some tasks. The results indicated that existing knowledge combined with the current findings, which take age into account, could be used in clinical settings to assess the kinematics of activities of daily living and set treatment goals.

## Graphical abstract

**Figure:**
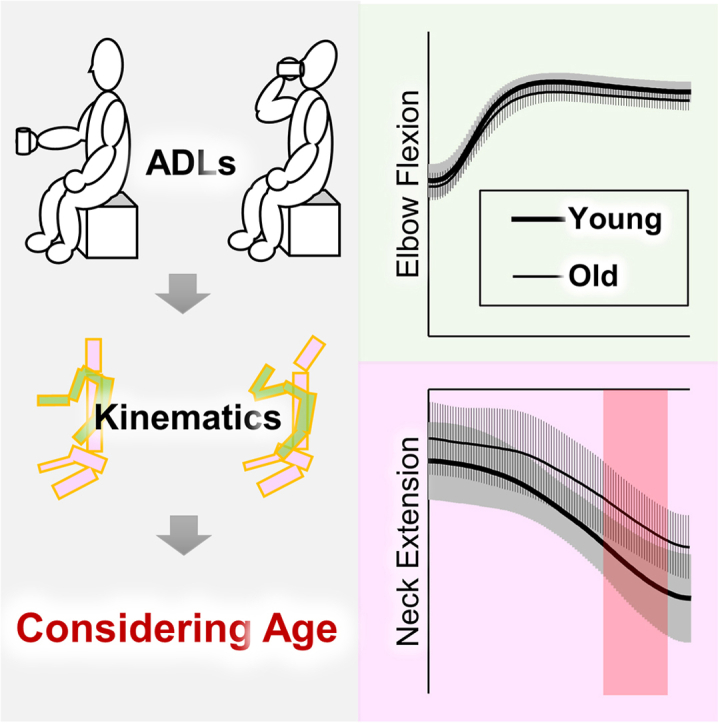

## 1 Introduction

Several studies have analyzed shoulder and elbow joint kinematics particularly in relation to reaching movements during activities of daily living (ADLs) in healthy adults [[1], [2], [3], [4], [5], [6], [7]], children [8,9], and patients with pathological conditions [10,11]. The ranges of motion (ROMs) of the shoulder and elbow when performing 66 ADLs, as investigated by Oostewijk et al. [12], are useful for clinicians because joint kinematics in healthy adults are used as a reference for evaluating patient conditions and setting rehabilitation and orthopaedic goals. In addition to upper limb movements, neck and trunk kinematics have been studied to identify realistic ADLs [[2], [3], [4], [5],^8^,^9^,^11^]. However, a recent review indicated that most studies include only younger adults [13]. Therefore, in an aging society, a clearer understanding of the effects of age on upper limb, neck, and trunk kinematics is required to provide better guidelines for rehabilitation and orthopedics.

Few studies have examined the effects of age on ADL kinematics in healthy adults; thus, limited understanding exists regarding reduced and slow motions caused by aging. In one study [14], compared with younger adults, older adults exhibited reduced shoulder flexion in tasks involving reaching for objects and body parts. This may be due to a nonuniform sex ratio between age groups. Decreased lumbar rotations during walking, climbing stairs, standing up, sitting down, and washing hair were predicted by the age factor [3]. Furthermore, drinking tasks took longer in older adults [15].

In contrast, older adults exhibited increased flexion angle at the shoulder joint during reaching [16] and switch pushing but not during drinking tasks [17]. Additionally, reduced neck motions during various ADLs were not predicted by the age factor [2]. Thus, we suspected that older adults have maintained or compensated large shoulder motions and maintained neck motions when performing ADLs.

Movements in ADLs vary because they include coincidental discrete hand reaching and combined motions of certain body segments; however, these conflicting and incomplete findings regarding ADLs in older individuals may have resulted from limited movement tasks or joint motions examined in each study. Existing kinematic information on ADLs in older individuals is difficult to understand and adapt to clinical settings.

Advanced age results in patients requiring assistance with ADLs [18,19]. Improving age-worsened kinematics through rehabilitation and orthopedics in this population is critical. Thus, we investigated the effect of healthy aging on clinically observable changes in comprehensive upper-body kinematics involving the upper limb, neck, and trunk during essential ADLs. These findings could help adapt human kinematics during ADLs to clinical practice for assessing and deciding goals for patients and engineering developments. On the basis of evidence that ADLs in older adults require a longer time and have fewer motions [3,14,15], we hypothesized that aging would affect upper-body kinematics and result in slower and decreased joint motions and smaller joint angle positions during ADLs. Therefore, we aimed to examine the in-depth differences in joint movements during essential ADLs between two age groups and determine the characteristics of ADL kinematics in healthy older adults.

## 2 Materials and methods

### 2.1 Participants

We recruited participants aged ≥20 years from the community-dwelling population, based on the following inclusion criteria: healthy individuals without musculoskeletal or neurological disorders requiring treatments or showing symptoms, those able to independently perform ADLs without assistance tools, those with no physical training for athletic competitions, and those able to understand the study protocol. These criteria were orally verified, with the participants attesting to meeting all the inclusion criteria. The participants provided written informed consent, and the protocol including anthropometry was approved by the Research Ethics Committee of the Faculty of Medicine of the University of Miyazaki (Miyazaki-shi, Japan; approval number O-0459). Each group had a required sample size of nine individuals, calculated with an effect size of d = 1.49, based on a previous study [16] that reported ROMs for shoulder elevation during a reaching task with low load in healthy younger and older adults (mean difference = 11.15°, standard deviations [SDs] = 6.33° and 8.45°, respectively) and a power of 0.8 using the *t*-test for detecting differences between two independent means with G*Power 3.1.9.7 (University of Düsseldorf, Düsseldorf, Germany). The younger adult (n = 12) and older adult (n = 10) groups were divided by age: 20–39 years and ≥60 years, respectively. We set two age interval groups because detecting differences in variables between serial age groups is difficult [20].

### 2.2 Motion capturing

The three-dimensional Vicon Nexus motion capture system (Oxford Metrics Ltd., Oxford, UK) was integrated with a full body Plug-In Gait Model (including the head, thorax, pelvis, two upper arms and two forearm segments, neck, trunk, and both shoulder joints with three degrees of freedom, and both elbow joints with one degree of freedom) [21] using a Butterworth filter with a 6-Hz cut-off. Twelve infrared cameras with sampling rates of 100 Hz were used. Thirty-five reflective markers were attached to the bilateral upper and lower extremities and the head, neck, trunk, and pelvis. This biomechanical model calculates joint angles from the relative position and direction of rigid body segments, which are obtained from the position of the attached markers and the anthropometric measurements of participants. The upper-body joint kinematics in this model have acceptable validity and reliability [5,22,23].

### 2.3 ADL tasks

**Fig. 1:**
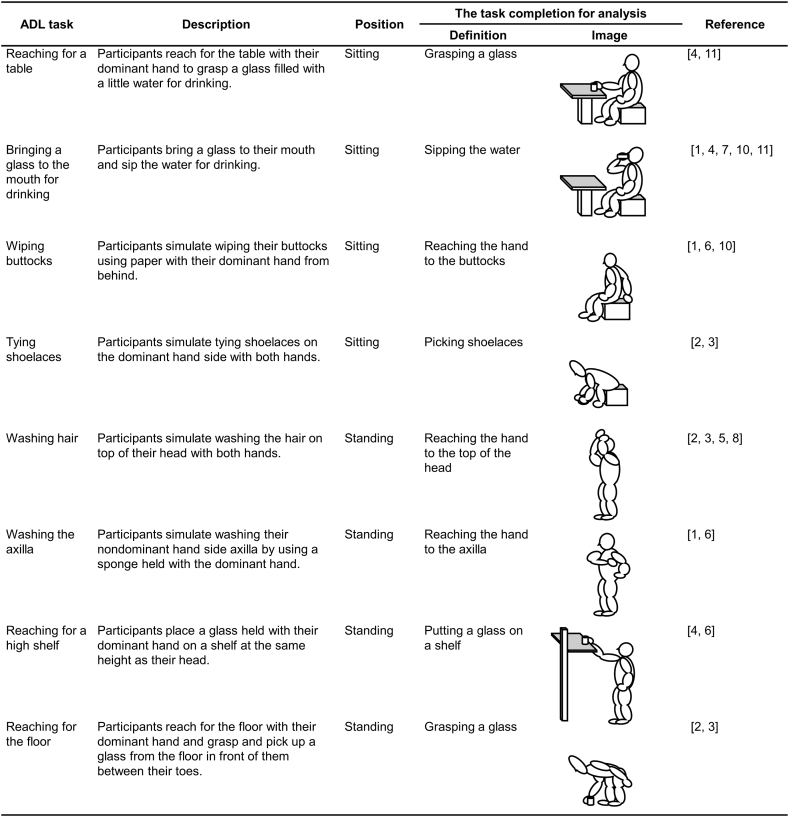
Definitions of the ADL tasks. ADL, activities of daily living.

We recorded movements during eight ADL tasks (Fig. 1) that included the requirements for independent daily living in older adults [24,25]. The drinking task was divided into two phases: reaching for a table and bringing a glass to the mouth for drinking. In the drinking task, a table was set in front of the individual at the length of the individual's thigh and height of the individual's elbow. In the “reaching for a high shelf” task, a table was set in front of the individual at arm's length and at the height of the top of the individual's head. When performing tasks in a sitting position, the individual started the task in a comfortable position on a stool, which was adjusted to the height of the individual's lower legs, with the hands on the thighs, except when bringing the glass to the mouth. For tasks in the standing position, individuals began the tasks with their upper limbs hanging comfortably. In the “reaching for a high shelf” task, they only held the glass with their dominant hand. The definitions and images of the task completion for analysis are shown in Fig. 1. Individuals performed each ADL four times at a self-selected speed; the first time was intended to familiarize the participants with the tasks.

### 2.4 Data processing

After visually inspecting the dominant hand reflective marker trajectory and motions of the stick figure while the participants performed the ADL tasks, joint angle data of the dominant shoulder flexion/extension, abduction/adduction, and internal/external rotation, elbow flexion/extension and neck and trunk flexion/extension, ipsilateral/contralateral flexion, and ipsilateral/contralateral rotation (all +/−) were derived from the biomechanical model. The dependent values were ROM (i.e., differences between the maximal and minimum joint angles), required time, and sequential joint angles from task initiation to completion. These were selected for measuring the ranges of joint motion, execution speed, and positions with time of the upper limb, neck, and trunk, respectively. Sequential joint angles were normalized with a 0–100% cycle time. These dependent values were the averages of values from the second, third, and fourth repetitions of each task for each individual.

### 2.5 Statistical analyses

To compare the groups, the ROMs and required time were analyzed using the independent Student *t*-test, Welch *t*-test, or Mann–Whitney *U* test, which were selected based on data normality and variance tests using JMP 16 (SAS Institute Inc., Cary, NC, USA) and calculated with effect size r [26]. Sequential joint angles were used for statistical parametric mapping (SPM) of two independent Student *t*-tests [27,28] to compare joint angle positions with the timing of movements between groups. The advantage of SPM is that entire continuous biomechanical data can be analyzed by calculating test statistics for the null hypothesis. The results can indicate periods of group differences in movement cycles in joint angle data of ADL tasks [9,11,16]. SPM was conducted using MATLAB R2021b (MathWorks, Inc., Natick, MA, USA) with an open-source code [29]. Three constituent null hypotheses were tested in this study; therefore, the statistical significance level was set at 1 - (1–0.05)^1/3^ = 0.017. Effect sizes (r) were small at 0.10, medium at 0.30, and large at 0.50 [26].

## 3 Results

### 3.1 Age group characteristics

The younger group included 12 individuals (7 men), 22–38 years old (29.8 ± 5.4 years); height, 1.64 ± 0.08 m; and weight, 60.7 ± 9.7 kg. The older group comprised 10 individuals (6 men), 60–77 years old (69.5 ± 4.9 years); height, 1.59 ± 0.10 m; and weight, 57.3 ± 10.2 kg. The sex ratio and anthropometry measurements between age groups were not significantly different (p ≥ 0.05), except for age (p < 0.0001). All individuals were right-handed in every task, except one older individual was right-handed only in performing the buttocks wiping task but left-handed in performing all other tasks.

### 3.2 ROMs and necessary times

**Fig. 2:**
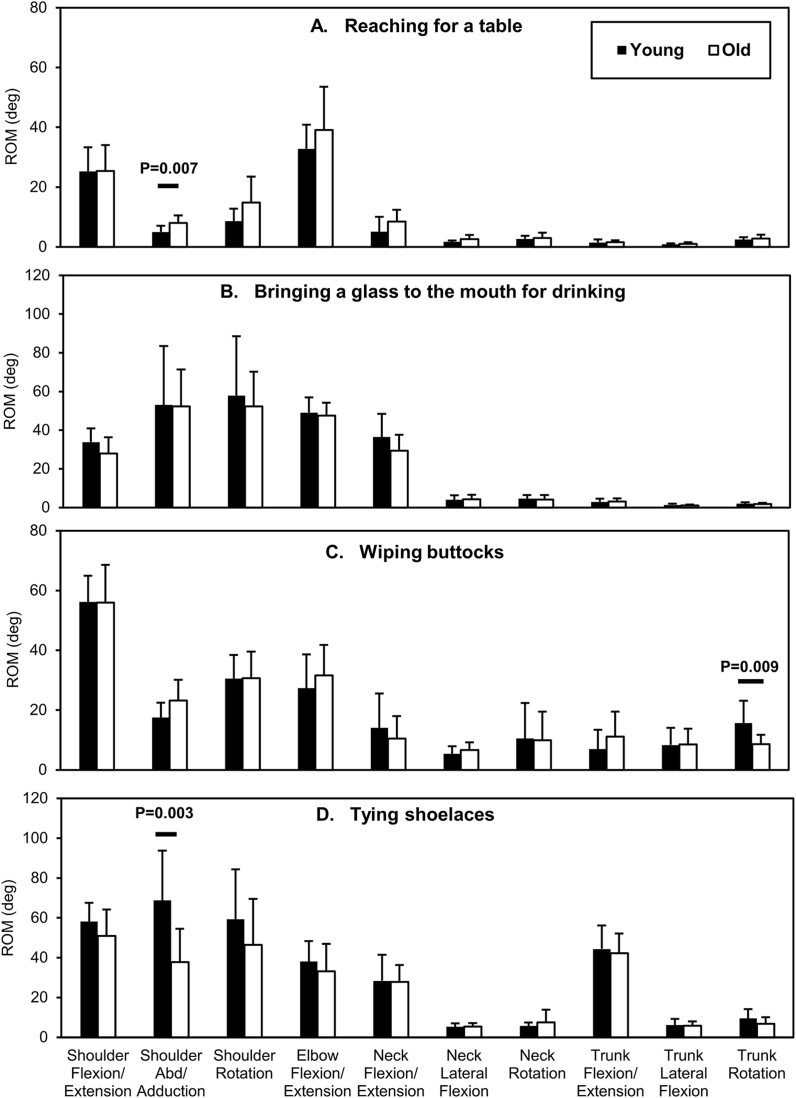
Comparison of ROMs between the age groups for tasks such as “reaching for table (A),” “bringing a glass to the mouth for drinking (B),” “wiping buttocks (C),” and “tying shoelaces (D).” The black and white bars indicate the means. The error bars indicate the SDs. Significant differences between the age groups (p < 0.017) are shown with the respective p-values. ROM, range of motion; SD, standard deviation.

**Fig. 3:**
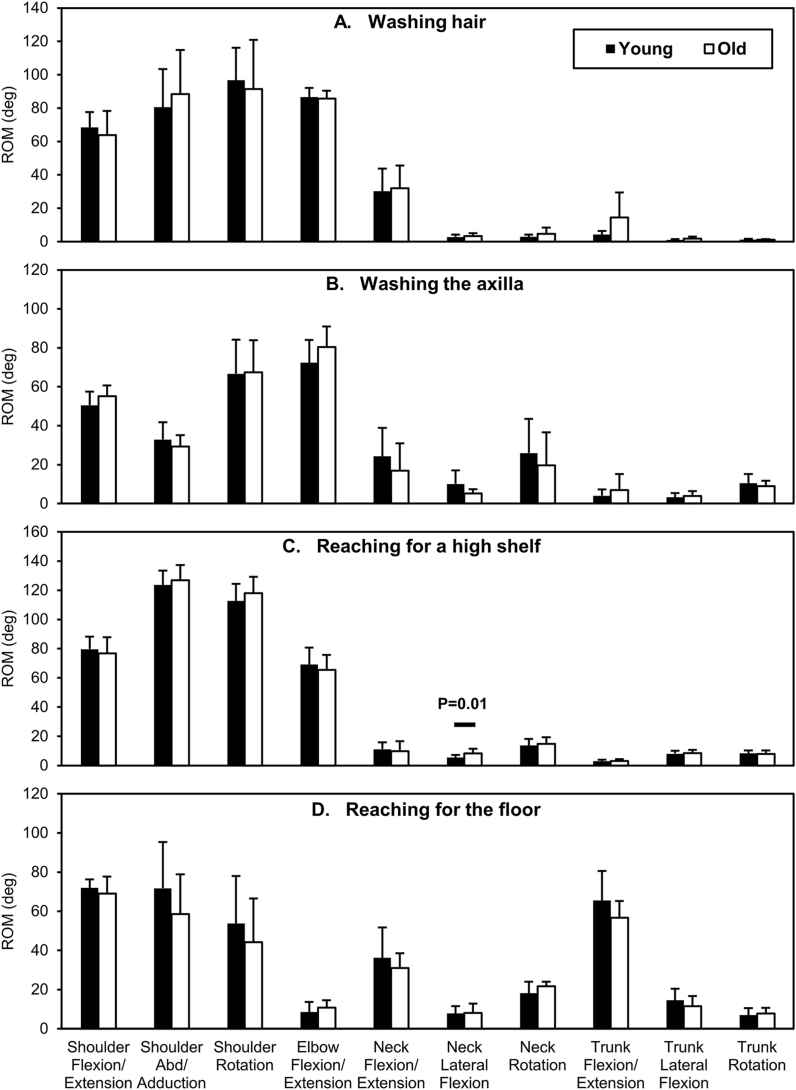
Comparison of ROMs between the age groups for tasks such as “washing hair (A),” “washing the axilla (B),” “reaching for a high shelf (C),” and “reaching for the floor (D).” The black and white bars indicate the means. The error bars indicate the SDs. Significant differences between the age groups (p < 0.017) are shown with the respective p-values. ROM, range of motion; SD, standard deviation.

The ROMs for the tasks are shown in Fig. 2, Fig. 3 (Supplementary Data 1 presents all ROMs with p-values and effect sizes). Four of 80 ROMs across eight tasks differed significantly between the two age groups. In the “reaching for the table” task, shoulder abduction/adduction was significantly greater in the older group than in the younger group with a large effect (8.0° ± 2.6° vs. 5.0° ± 2.1°, p = 0.007, r = 0.56). In the “buttock wiping” task, the older group, compared with the younger group, exhibited significantly smaller trunk ipsilateral/contralateral rotation with a large effect (8.6° ± 3.1° vs. 15.7° ± 7.4°, p = 0.009, r = 0.53). The older group exhibited significantly less shoulder abduction/adduction than did the younger group in the “tying shoelaces” task with a large effect size (37.7° ± 16.7° vs. 68.8° ± 24.9°, p = 0.003, r = 0.60). In the “reaching for a high shelf” task, the older group, compared with the younger group, exhibited significantly greater ipsilateral/contralateral flexion of the neck with a large effect (8.3° ± 3.0° vs. 5.4° ± 1.8°, p = 0.01, r = 0.54). The elbow flexion ROMs in all tasks, the necessary times for all ADLs (Table 1), and all ROMs in the “drinking,” “washing hair,” “washing the axilla,” and “reaching for the floor” tasks did not differ significantly and did not have a large effect (p ≥ 0.017, r < 0.50).

**Table 1:** The time required to perform ADLs in each age group.

ADL task	Young		Old	
	Mean ± SD	95% CI	Mean ± SD	95% CI
Reaching for a table	1.00 ± 0.23	0.86–1.15	1.17 ± 0.21	1.02–1.32
Bringing a glass to the mouth for drinking	2.03 ± 0.34	1.81–2.24	2.42 ± 0.60	1.99–2.85
Wiping buttocks	1.54 ± 0.31	1.34–1.73	1.38 ± 0.20	1.24–1.53
Tying shoelaces	1.68 ± 0.23	1.54–1.82	1.78 ± 0.36	1.53–2.04
Washing hair	1.10 ± 0.29	0.91–1.28	1.13 ± 0.19	1.00–1.27
Washing the axilla	1.05 ± 0.17	0.95–1.15	1.04 ± 0.13	0.95–1.14
Reaching for a high shelf	1.99 ± 0.46	1.70–2.29	2.28 ± 0.32	2.05–2.51
Reaching for the floor	1.43 ± 0.18	1.32–1.54	1.48 ± 0.19	1.35–1.62

The time unit is in seconds. The ages of the younger and older groups (presented as the mean ± SD) are 29.8 ± 5.4 years and 69.5 ± 4.9 years, respectively.

ADL, activities of daily living; SD, standard deviation; CI, confidence interval.

### 3.3 Sequential joint angles

**Fig. 4:**
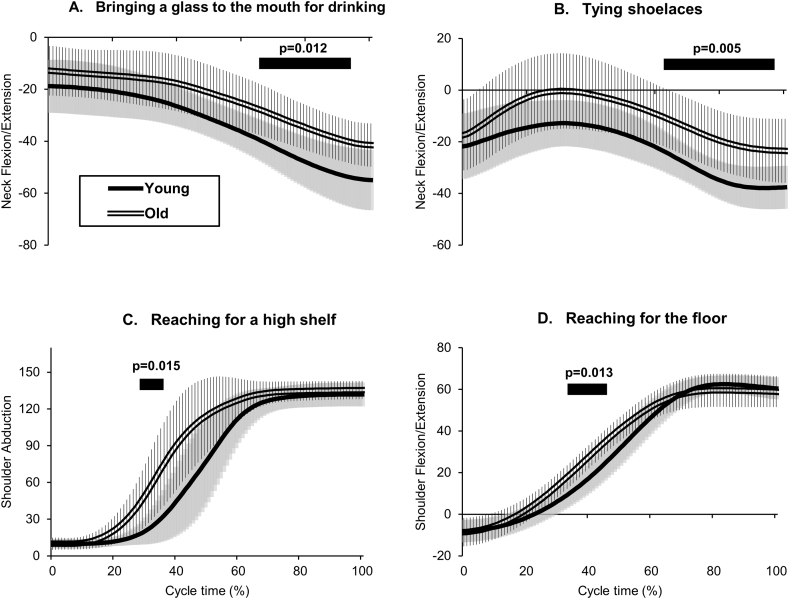
Sequential joint angles with significant differences in ADL tasks. A, bringing a glass to the mouth for drinking; B, tying shoelaces; C, reaching for a high shelf; D, reaching for the floor. The vertical axis indicates joint angles (degrees) in each labeled joint motion. Single and double lines indicate the mean values. Gray and vertical striped areas indicate the SDs. The black horizontal bars indicate durations in cycle times; significant differences between age groups were evaluated by SPM (p < 0.017). ADL, activities of daily living; SD, standard deviation; SPM, statistical parametric mapping.

Fig. 4 shows significant differences between the groups for 80 motions across eight tasks in four sequential joint angles normalized with 0–100% of the task duration (Supplementary Data 2 presents all continuous joint angles). The older group exhibited reduced neck extension in the “bringing a glass to the mouth for drinking” task during the latter half of the task (67–92% of the duration, p = 0.012). In the “tying shoelaces” task, the older group demonstrated less neck extension (64–95%, p = 0.005). The older group had greater angles for shoulder abduction in the “reaching for a high shelf” task (30–34%, p = 0.015) and shoulder flexion in the “reaching for the floor” task (35–44%, p = 0.013). Elbow flexion and trunk angles did not differ significantly between the older and younger groups across all tasks (p ≥ 0.017). Joint angles in “reaching for a table,” “wiping buttocks,” “washing hair,” and “washing axilla” tasks were also not significantly different between the groups (p ≥ 0.017).

## 4 Discussion

### 4.1 Main findings

This study examined the clinically observable kinematic differences in the shoulder, elbow, neck, and trunk during basic ADLs between healthy younger and older adults. Our results showed that most joint kinematics, including elbow-related variables in all tasks in older individuals, were maintained, which contradicted our hypothesis. However, some altered kinematics partly affirmed our hypothesis. Older adults had reduced joint motions of the shoulder in the “tying shoelaces” task and of the trunk in the “wiping buttock” task. The analyzed sequential angle data indicated that aging exerted an effect on smaller neck joint angle positions in the latter phases of “bringing a glass to the mouth for drinking” and “tying shoelaces” tasks.

Some factors regarding the results should be considered. This study aimed to obtain clinically useful data, which required easy-to-distinguish changes due to age. The sample size was accordingly planned for detecting large effect sizes of d ≥ 0.8 [26], based on substantial values previously reported [16]. Additionally, if kinematic variables continuously change with age, then our comparison of two generations with age intervals could aid in detecting these differences. Our findings indicated large effects in group differences, whereas variables with no differences indicated mostly none to medium effects. The findings of no differences between groups suggested that these kinematic variables were maintained with possible small-to-medium effects between the groups. Such variables with no differences constituted most of the joint angles investigated (76/80 ROMs and 76/80 sequential joint angles); therefore, we inferred that no effect of healthy aging existed in the upper-body kinematics of ADLs.

### 4.2 Kinematics maintained in older adults

In previously reported ADL-related tasks comprising reaching the mouth, top of the head, or an object in front of the participant, older adults had smaller maximal shoulder flexion and no differences in elbow flexion angles than did younger adults [14]. However, the study design of the previous study was limited by its nonuniform sex ratio—the young group consisted only of men, whereas men comprised 50% of the older group. The differences in the shoulder angle in the previous study may have been caused by the age and/or sex factor, which was addressed in this study by having a similar sex ratio between the younger and older groups. Herein, the sequential data of shoulder and elbow joint angles in the “bringing a glass to the mouth for drinking,” “reaching for a table,” and “washing hair” tasks, which were similar to tasks performed in the previous study, did not differ significantly between the groups. In our study, ROMs and sequential joint angles of the shoulder and elbow flexion were maintained when older individuals performed these three tasks. The elbow joint motion ranges and angle positions were maintained in every ADL task presented in this study. This finding suggests the influence of sex on kinematics in ADLs; therefore, future studies should consider this factor or conduct a sex-segregated study using a larger sample.

Furthermore, older individuals required a longer time for the drinking task than did younger individuals [15]; however, our results showed a comparable or somewhat longer time in the older group with no significance, except in the medium effect sizes. The different findings between the studies may be due to the large age gap between groups in the previous study, compared with the small age gap in this study. A future comparison study of speed between age groups with gradation should be considered.

### 4.3 Factors affecting kinematics

In this study, older adults exhibited reduced ROMs in trunk rotation and shoulder abduction/adduction during “wiping buttocks” and “tying shoelaces,” respectively. In the “drinking” and “tying shoelaces” tasks, the neck extension angle was smaller in the older group during the cycle, and the differences were significant in the latter half of the cycle durations. One factor that may cause reduced motion and angle position in older individuals is muscle strength, which decreases in the whole body with age [30]. An important factor to mention is that older individuals, compared with younger individuals, do not require greater muscle effort in light-load ADLs but do require it in heavy-load ADLs [31]. This finding may explain why older individuals showed smaller angles in some but not all ADL tasks; joint motions for most tasks in this study required less muscle strength in older individuals. Age differences may have caused differences in voluntary maximal ROMs between age groups in parts of kinematics during ADLs. Both groups exhibited higher neck extension in the “drinking” task than in other tasks, which differed significantly between the groups. These angle values were nearly identical to the voluntary maximal ROMs previously reported, which also differed significantly between groups [32,33]. However, the positions of most joint angles of the upper body during essential ADLs, which were not affected by age, may be lower than the maximal ROMs. Thus, the joint motion and angle position during the current ADLs may be maintained in older adults.

Older individuals exhibited lower ROMs in shoulder abduction/adduction in the “tying shoelaces” task when participants needed to reach toward a lower position; however, the ROMs increased in the “reaching for the table” task, which resisted gravity. For upper limb elevation against gravity, larger shoulder motions may have had the same propensity as the greater shoulder flexion position in forward switch pushing in older adults [13]. These movements in older adults in relation to vertical gravity possibly compensate for the reductions in time and energy [20]. Our findings of some larger and smaller shoulder motions may explain the movement strategy of older individuals.

Sequential joint angle analysis revealed that shoulder abduction and shoulder flexion in “reaching for a high shelf” and “reaching for the floor” tasks, respectively, increased sooner in older individuals than in young individuals. This timing appeared consistent with established upper limb kinematics in which the ratio of primary submovement to total movement is lower in older individuals [34]. The rapid movement warrants activation of the upper trapezius [35] and induces the large ROM of neck lateral flexion observed in the “reaching for a high shelf” task. The aforementioned altered ADL kinematics of the joint level in older individuals, which may rarely occur in clinical settings, may be healthy age-related changes. In future studies, this may be more noticeable in adults older than those in our older group.

### 4.4 Clinical implications

In clinical practice, measurement data for static ROMs obtained with goniometers and/or movements during ADL tasks recorded with motion capture systems are used to evaluate patients’ conditions. Existing knowledge regarding the kinematics of ADLs in healthy adults [[1], [2], [3], [4], [5], [6], [7],^12^], which indicates ranges and peak values of joint motion and motion patterns, could be used as a reference because age does not affect most of these variables. As a cause of deviations from reference ranges, the effect of age could also be considered using our results. Elbow joint kinematics in essential ADLs in older adults and most other upper-body joint angles should be maintained and set at preferential goals.

We examined the shoulder, elbow, neck, and trunk kinematics comprehensively during essential ADL tasks for older individuals, and thus addressed the inconsistent partial knowledge derived from previous studies [2,3,[14], [15], [16], [17]]. The study findings can be integrated into existing literature on ADL kinematics and will enable kinematic assessments and the formulation of goals for crucial ADLs in older patients. Moreover, the upper-body joint kinematics addressed in the current study are influenced by flexibility or stability; therefore, muscle strength, sensations, learned motion patterns, intervention measures in rehabilitation, orthopedics, and engineering are important for reaching kinematic goals during ADLs. Future studies on human kinematics during ADLs that consider the effect of age should be conducted.

### 4.5 Limitations

Variables in some tasks such as ROMs of neck flexion and rotation in the “wiping buttocks” task showed large standard deviations. These variances may reflect the existence of self-selected movement strategies such as looking or not looking at their buttocks. The investigated tasks did not require high muscle strength or near-full ROMs such as carrying a heavy load or reaching for the middle of the back, which may affect ADL kinematics and tasks requiring high speed. A limited set of tasks were considered, and effects of a job, exercise habits, and eye disorders or others were not included. The kinematics of hand segments or lower extremities were also not examined. Each age group had some age disparities that may not have demonstrated kinematic differences. This study included a small sample size; therefore, further research with a large sample size is needed to validate our results.

## 5 Conclusions

The analysis of eight ADL tasks indicated that, except in some parts of movements, healthy older adults mostly maintain the joint kinematics of their upper extremity, neck, and trunk. Elbow kinematics across all examined tasks were unaffected by age. Decreased movement speed in older adults was also not evident. The effects of age on altered kinematics were characterized by reduced shoulder and trunk motions and reduced neck joint extension angles in the latter phases of movements or caused earlier upper limb elevation. Greater ranges of upper limb elevation in the direction of gravity were observed in the “reaching for a table” task. This study's findings may improve established knowledge of kinematics during ADLs for clinical evaluation, choice of goals for patients, and development of engineering solutions. In future studies, age groups with gradations and sex should be analyzed.

## Ethics statement

This study was reviewed and approved by the Research Ethics Committee of the Faculty of Medicine of the 10.13039/501100006452University of Miyazaki, Miyazaki-shi, Japan (approval number: O-0459). All participants provided informed consent to participate in the study.

## Data availability statement

The research data are confidential.

## CRediT authorship contribution statement

**Jun Nakatake:** Writing – review & editing, Writing – original draft, Validation, Methodology, Investigation, Formal analysis, Data curation, Conceptualization. **Hideki Arakawa:** Writing – review & editing, Validation, Funding acquisition. **Maeda Shogo:** Validation, Methodology, Investigation, Data curation, Conceptualization. **Koji Totoribe:** Writing – review & editing, Resources. **Etsuo Chosa:** Writing – review & editing, Validation, Supervision, Project administration, Conceptualization.

## Declaration of competing interest

The authors declare that they have no known competing financial interests or personal relationships that could have appeared to influence the work reported in this paper.
